# Autopsy-Based Analysis of Landmine Blast Fatalities Associated With Left-Wing Extremism

**DOI:** 10.7759/cureus.105953

**Published:** 2026-03-27

**Authors:** Sawan Mundri, Nityanand Kumar, Anand Kumar, Kumar Shubhendu, Ankur Chaudhary, Ajay K Bhagat

**Affiliations:** 1 Department of Forensic Medicine and Toxicology, Rajendra Institute of Medical Sciences, Ranchi, IND; 2 Department of Forensic Medicine and Toxicology, Laxmi Chandravansi Medical College and Hospital, Palamu, IND; 3 Department of Forensic Medicine and Toxicology, Hi-Tech Medical College and Hospital, Rourkela, IND

**Keywords:** blast injuries, hemorrhagic shock, improvised explosive devices, landmines, left wing extremism

## Abstract

Background

The utilization of landmine detonations, frequently facilitated via improvised explosive devices (IEDs), continues to be a favored strategy employed by Maoist insurgents in regions of India afflicted by left-wing extremism (LWE). Personnel tasked with anti-insurgency operations face an elevated probability of incurring fatal blast-related injuries. Conducting a forensic analysis of such fatalities is crucial to establishing consistent patterns of injury, enhancing protective measures, and guiding medico-legal protocols.

Methodology

A retrospective analysis was conducted at the Department of Forensic Medicine and Toxicology, Rajendra Institute of Medical Sciences, Ranchi, Jharkhand. Autopsy records of six security personnel who sustained fatal injuries in a landmine blast during an anti-Naxalite operation in Jharkhand were reviewed. External and internal injuries, patterns of anatomical involvement, and immediate causes of death were systematically documented and analyzed.

Results

All individuals who succumbed were male, with their ages spanning from 27 to 36 years. The significant injuries noted comprised traumatic amputations of the legs, major lacerations, and fractures that were comminuted. Injuries were predominantly localized to the inferior segment of the body, which aligns with the mechanics associated with landmine detonation. In every recorded instance, death was attributed to hemorrhagic shock in conjunction with polytrauma. Standard-issue protective equipment demonstrated a significant lack of efficacy in mitigating injuries resulting from high-energy secondary and tertiary blast forces.

Conclusion

This study reveals a consistent and characteristic pattern of injuries in landmine blast fatalities among security personnel in Maoist-affected regions. The findings underscore the need for enhanced forensic documentation, targeted improvements in personal protective equipment, and refined tactical protocols. Multidisciplinary efforts combining forensic insights, trauma care, and strategic planning are vital to reducing operational casualties in insurgency zones.

## Introduction

Landmines and improvised explosive devices (IEDs) are widely used in modern asymmetric conflicts because of their capacity to cause severe injuries and fatalities. These concealed explosive devices may be activated by pressure, proximity, or remote detonation and are designed to produce high-energy blast waves along with the projection of fragments and debris [1]. When detonated, landmine explosions produce complex patterns of trauma characterized by extensive soft-tissue destruction, skeletal fractures, and traumatic amputations, particularly among individuals positioned close to the point of detonation [2].

Blast-related injuries are commonly classified into four categories based on the mechanism of injury: primary injuries caused by the direct effects of blast overpressure on gas-containing organs such as the lungs and intestines, secondary injuries resulting from high-velocity fragments or debris, tertiary injuries occurring when victims are displaced by the blast wind and strike surrounding objects, and quaternary injuries that include burns, inhalational injuries, and other associated effects [3-5]. Fatal blast incidents frequently involve a combination of these mechanisms, leading to severe polytrauma. In such cases, medico-legal autopsy plays an essential role in identifying the precise cause of death, reconstructing the dynamics of the explosion, and documenting characteristic injury patterns relevant to forensic investigations.

In India, landmine and IED attacks are frequently associated with the long-standing insurgency related to left-wing extremism (LWE), which predominantly affects the central and eastern regions of the country. Several states, including Jharkhand, Chhattisgarh, Odisha, and Maharashtra, have experienced recurrent insurgent attacks in which explosive devices are strategically placed along forest routes, patrol tracks, or vehicular pathways to target security forces engaged in counter-insurgency operations [6]. According to reports from the South Asia Terrorism Portal (SATP), Maoist-related violence has resulted in thousands of fatalities among civilians and security personnel since 2000, with explosive devices responsible for a substantial proportion of these deaths [7].

Despite the recurrent occurrence of such incidents, systematic forensic analyses of landmine blast fatalities in India remain limited, particularly in regions affected by LWE. Detailed documentation of autopsy findings in such cases is essential for understanding injury patterns, mechanisms of death, and the potential influence of protective equipment or operational factors on fatal outcomes [8,9]. Furthermore, region-specific forensic evidence can inform evidence-based policy recommendations for protective equipment design, operational tactics, and medical preparedness in conflict-affected environments.

Therefore, the present study aimed to systematically analyze the autopsy findings, injury patterns, and mechanisms of death in landmine blast fatalities associated with LWE in an affected region of India, with a view to generating forensic evidence that may contribute to improved medico-legal documentation and enhanced protective strategies for personnel operating in high-risk environments.

## Materials and methods

Performed in the Forensic Medicine and Toxicology (FMT) Department of the Rajendra Institute of Medical Sciences (RIMS) in Ranchi, Jharkhand, India, the inquiry principally analyzed the autopsy records concerning security personnel who died from damage caused by a landmine explosion during an anti-Naxalite operation. The criteria for inclusion encompassed confirmed fatalities that were a direct outcome of a singular, meticulously documented landmine detonation occurring within a specified operational context, with comprehensive autopsy records accessible for examination. This investigation specifically encompassed cases involving security personnel who were present at the site of the explosion and whose deaths could be unequivocally linked to the detonation based on both medico-legal and operational evidence.

Any deaths that could not be clearly linked to the specific landmine explosion in question, including those caused by unrelated injuries, existing health issues, or environmental influences after the blast, were left out of the study. Additionally, cases involving individuals who were not in direct proximity to the explosion (for instance, personnel who died at a remote location or after a significant delay due to subsequent complications) were also excluded. Moreover, victims characterized by ambiguous circumstances or compounded trauma from concurrent external events (such as secondary attacks or unrelated operational risks) were excluded, in order to preserve the study's emphasis on direct and immediate fatal consequences stemming from a singular, well-documented explosive incident.

Comprehensive data were meticulously extracted from the autopsy reports, including both external and internal examination results. The data collected included demographic variables (age and sex), occupational and operational details, and mode of movement, along with the patterns and anatomical distribution of injuries and wound characteristics, including lacerations, fractures, and traumatic amputations. When accessible, data concerning the use of personal protective equipment (PPE) during the occurrence of injury were also documented to evaluate its efficacy. Injuries were categorized in accordance with established forensic blast injury classifications. This classification system enabled a methodical examination of the mechanisms and severity of injuries.

This research was undertaken in strict conformity with the ethical standards that regulate studies involving the deceased, reflecting a steadfast commitment to confidentiality and the utmost respect for both the departed individuals and their mourning families. Data pertaining to demographics and injuries were captured through the application of descriptive statistical techniques. Patterns of injury were analyzed to identify consistent features across the cases, with the aim of improving forensic documentation, guiding medico-legal investigations, and informing preventive and protective strategies in similar operational contexts.

## Results

This research investigated the comprehensive autopsy results of six deceased male security personnel who sustained fatal injuries resulting from a singular landmine detonation during a counter-insurgency operation within a forested area of Jharkhand afflicted by Maoist activity. The ages of those who have died varied from 27 to 36 years, averaging out to 31.2 years. All individuals were assigned to active field duty and were traversing in a fortified personnel carrier that was specifically targeted by the landmine (Table 1). The explosion transpired unexpectedly, indicating a high level of expertise in concealment and the potential implementation of anti-handling devices.

**Table 1:** Demographic and operational characteristics of the victims

Case No.	Age (years)	Sex	Occupation	Operation type	Mode of movement
1	27	Male	Security personnel	Patrol	Vehicle
2	28	Male	Security personnel	Patrol	Vehicle
3	29	Male	Security personnel	Patrol	Vehicle
4	36	Male	Security personnel	Patrol	Vehicle
5	27	Male	Security personnel	Patrol	Vehicle
6	27	Male	Security personnel	Patrol	Vehicle

Circumstantial evidence suggested that the apparatus in question was a pressure-sensitive IED, concealed beneath an unpaved thoroughfare in a jungle environment. The intensity of the explosion was adequate to entirely obliterate the vehicle, which was forcefully elevated and thrust into the atmosphere for several meters, resulting in the ejection of certain personnel from within. The subsequent collision of the vehicle upon its return to the terrestrial surface also resulted in catastrophic injuries. Reports indicate that all six individuals perished due to their injuries at the incident site, before any medical help could arrive. Each of the individuals who died was donned with typical protective gear, including helmets for ballistic defense, body armor, and tactical boots. Nevertheless, the injuries incurred were predominantly fatal, despite the utilization of such protective gear.

External examination revealed catastrophic and mutilating injuries, particularly affecting the lower body. All victims except one had sustained traumatic lower-limb amputations: three had bilateral amputations, mostly at or above the knee, while two had a unilateral amputation with extensive injuries to the opposite limb. The amputations exhibited irregular, shredded wound margins and exposed bone with severe avulsion of soft tissues. Massive hemorrhage was evident in all cases. Additional injuries included multiple bone-deep lacerated wounds over the anterior aspect of the neck in one case, bone-deep bilateral upper limb lacerations in three cases, bone-deep unilateral upper limb lacerations in one case, evisceration of abdominal viscera in one case resulting from a large cavity deep laceration, and extensive lacerations in the groin, perineum, and lower torso in two cases. Flash burns and thermal injuries were present in four cases. Extensive areas of blackening were evident in two cases (Table 2).

**Table 2:** Autopsy profile of blast-induced injuries in security personnel

Case No.	Singeing of hairs	Blackening	Gunpowder residues	Splinters/foreign material	Number of lacerated wounds	Size and depth of the lacerated wounds	Location of lacerated wounds	Tissues affected	Fractures	Separated/missing body parts
1	Present over left thigh and right leg	Present over lower part of left thigh	Absent	Dirt particles	2	13 cm × 4 cm × bone deep	Left thigh	Soft tissues, blood vessels, bones	Left femur, right tibia, right fibula	Lower part of right leg and foot
						18 cm × 10 cm × bone deep	Right leg			
2	Present over right thigh	Absent	Absent	Glass splinter, dirt particles	4	12 cm × 8 cm × bone deep	Neck	Soft tissues, blood vessels, trachea, esophagus, bones + scrotum/testes	Right maxilla, mandible, right tibia, right fibula, left tibia, left fibula	Distal part of left foot
						5 cm × 4 cm × soft tissue deep	Right thigh			
						30 cm × 15 cm × bone deep	Right thigh + right leg			
						35 cm × 12 cm × bone deep	Left leg			
3	Absent	Absent	Absent	Cloth fragment, dirt particles	3	5 cm × 4 cm × bone deep	Left forearm	Soft tissues, blood vessels, bones	Left radius, left ulna, right femur	None
						15 cm × 8 cm × soft tissue deep	Left thigh and knee			
						10 cm × 4 cm × bone deep	Right thigh			
4	Present on both thighs	Absent	Absent	Dirt particles	7	5 cm × 2 cm × soft tissue deep	Right forearm	Soft tissues, blood vessels, bones + scrotum/testes	Left femur, right tibia, right fibula	Distal part of left thigh and leg, lower part of right leg and foot
						6 cm × 2 cm × soft tissue deep	Left forearm and elbow			
						8 cm × 2 cm × soft tissue deep	Scrotum			
						15 cm × 10 cm × soft tissue deep	Left thigh			
						12 cm × 4 cm × bone deep				
						20 cm × 15 cm × soft tissue deep	Right thigh			
						13 cm × 4 cm × bone deep	Right leg			
5	Absent	Absent	Absent	Cloth fragment, dirt particles	4	9 cm × 5 cm × bone deep	Left elbow	Soft tissues, blood vessels, bones	Left radius, left ulna, left tibia, left fibula, right radius, right ulna, right femur	Lower part of left leg and foot, distal part of right thigh and leg
						45 cm × 10 cm × bone deep	Left thigh and leg			
						10 cm × 8 cm × bone deep	Right forearm and elbow			
						20 cm × 15 cm × bone deep	Right thigh			
6	Present over right thigh and pubic region	Absent	Present over right anterior aspect of lower part of abdomen	Cloth fragment, dirt particles	6	15 cm × 10 cm × bone deep	Left forearm and palm	Soft tissues, blood vessels, bones, intestines, mesentery, omentum, penis, scrotum/testes	Left radius, left ulna, small bones of left palm and right palm, left femur, right femur	Distal part of left thigh and leg, distal part of right thigh and leg
						18 cm × 10 cm × bone deep	Right forearm and palm			
						35 cm × 30 cm × soft tissue deep	Abdomen			
						45 cm × 15 cm × bone deep	Left thigh and leg			
						10 cm × 5 cm × soft tissue deep	Penis, scrotum, and testis			
						25 cm × 15 cm × bone deep	Right thigh			

Internal examination findings indicated the presence of polytrauma consistent with both secondary and tertiary blast dynamics. The skeletal injuries observed included one case with facial bone fractures, three cases featuring long-bone fractures in the upper limb, one instance of hand small bone fractures, and long-bone fractures of the lower limb across all cases. Documentation revealed lacerations involving the carotid vessels, trachea, and esophagus in one particular case. In one case, embedded glass fragments were extracted, while pieces of fabric were discovered deeply embedded within lacerated wounds in three instances. Soil and sand particles were also identified as being entrenched within several lacerated wounds across all cases. Notably, fractures of the skull or occurrences of intracranial hemorrhages were markedly absent (Table 2).

Case descriptions

Case 1

**Figure 1:**
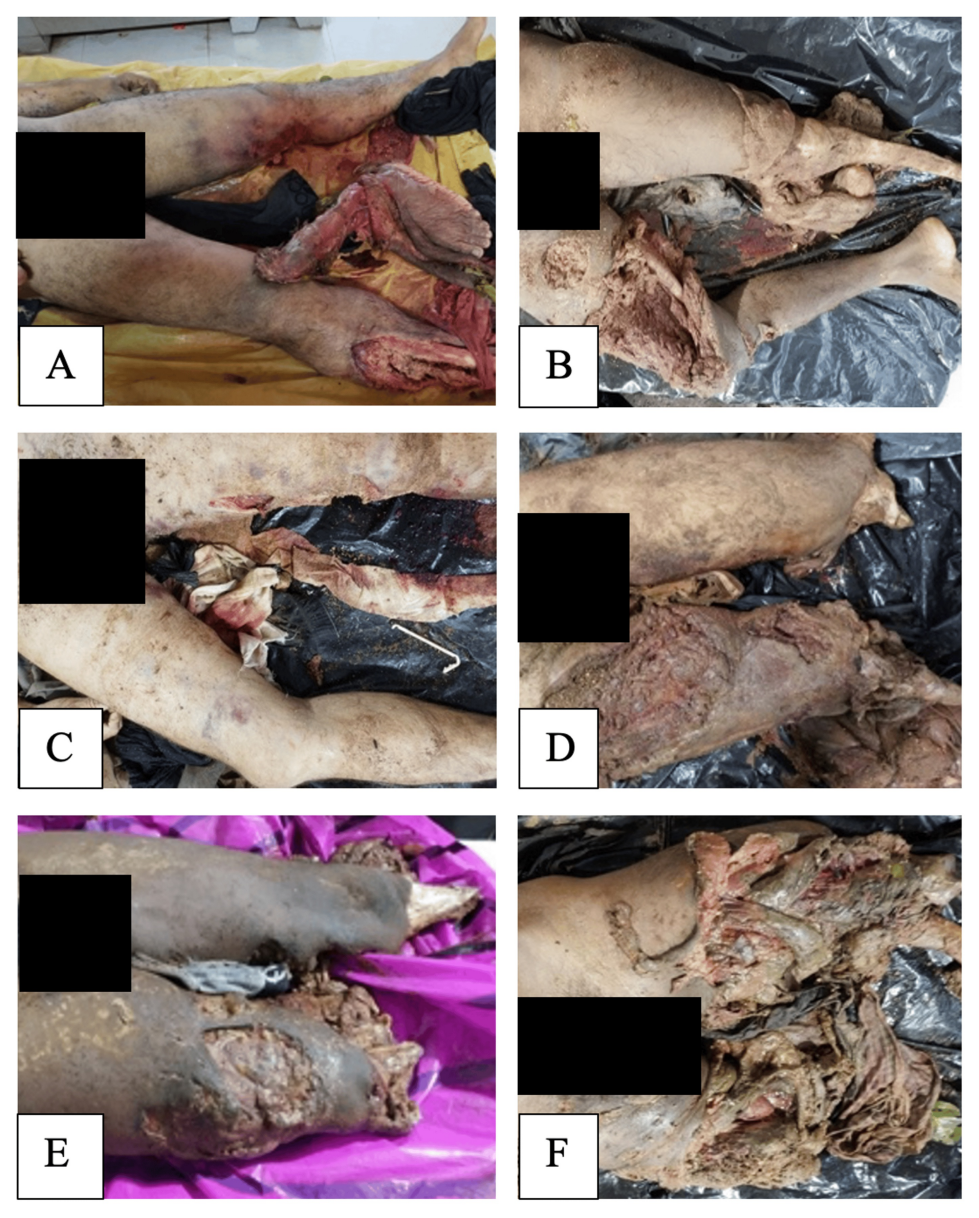
Postmortem imaging of the cases showing extensive lower-limb trauma (A) Traumatic blast injury of the lower limbs showing bone-deep lacerations with fractures of the femur, tibia, and fibula, and distal right limb separation in Case 1. (B) Extensive lower-limb blast injuries with bone-deep lacerations and traumatic loss of the distal left foot segment in Case 2. (C) Lacerated wounds over the thighs with an underlying right femoral fracture consistent with blast-induced trauma in Case 3. (D) Severe lower-limb blast injuries with bone-deep lacerations and traumatic separation of distal limb segments in Case 4. (E) Extensive lacerated wounds of the thigh and leg with associated long-bone fractures and distal limb loss in Case 5. (F) Bilateral lower-limb blast injuries with extensive lacerations, femoral fractures, and traumatic limb separation in Case 6.

A 27-year-old male sustained severe blast injuries predominantly involving the lower limbs. Two major bone-deep lacerations were present. One was located over the posterior aspect of the lower part of the left thigh, extending to the adjacent knee, resulting in fracture of the distal femur. Another extensive laceration over the anteromedial aspect of the right leg caused fractures of the distal tibia and fibula with traumatic separation of the distal limb segment (Figure 1A). Singeing of hair and localized blackening were observed over the left thigh and right leg.

Case 2

A 28-year-old male exhibited multiple severe blast injuries. Extensive bone-deep lacerations were present over the anteromedial aspect of the right thigh and adjoining right leg, associated with fractures of the proximal tibia and fibula. A large lacerated wound over the anterior aspect of the left leg resulted in traumatic loss of the distal foot segment (Figure 1B). Additional injuries included a deep laceration of the anterior neck involving major vessels and aerodigestive structures, and lacerations of the scrotum and testes. A glass splinter was recovered from the right groin region.

Case 3

A 29-year-old male sustained multiple blast-related injuries involving the extremities. A soft-tissue deep laceration over the posterior aspect of the left thigh extending to the adjoining knee and a bone-deep laceration over the posterior aspect of the right thigh resulted in fracture of the femur shaft (Figure 1C). Additional injuries included a bone-deep laceration over the left forearm with fractures of the radius and ulna. A cloth fragment embedded within one of the wounds was recovered.

Case 4

A 36-year-old male sustained extensive blast trauma involving both upper and lower extremities and the genital region. Multiple lacerations were present over the thighs and legs. A bone-deep laceration over the anterior aspect of the lower part of the left thigh resulted in fracture of the distal femur with traumatic loss of the distal limb segment. Another bone-deep laceration over the anterior aspect of the upper part of the right leg caused fractures of the proximal tibia and fibula with separation of the distal segment (Figure 1D).

Case 5

A 27-year-old male sustained severe blast injuries affecting both upper and lower limbs. A large, bone-deep lacerated wound extending from the posterior aspect of the left thigh to the proximal part of the left leg resulted in fractures of the proximal tibia and fibula with loss of the distal limb segment. Another bone-deep laceration over the anteromedial aspect of the right thigh was associated with fracture of the femur shaft and distal limb separation (Figure 1E). Additional injuries included fractures of both forearms. Cloth fragments were recovered from the wounds.

Case 6

A 27-year-old male sustained extensive blast injuries involving the extremities, abdomen, and genital region. Extensive bone-deep lacerations over both thighs resulted in fractures of the distal femurs with traumatic separation of distal limb segments (Figure 1F). Additional injuries included lacerations of both forearms with fractures of the radius, ulna, and hand bones, as well as a large abdominal wound with evisceration. Gunpowder residue and singeing of hair were observed over the abdomen and upper thigh.

In all six cases, death was attributed to hemorrhagic shock resulting from severe polytrauma. Massive vascular injury led to rapid exsanguination. The fatal injuries were overwhelmingly concentrated in the lower body, consistent with the mechanism of an upward-directed landmine blast. PPE analysis revealed limited efficacy in mitigating fatal injuries. While ballistic helmets and torso armor provided some protection to the upper body and likely prevented more extensive cranial trauma, the PPE used for the lower limbs was insufficient against the upward force of the explosion. In most cases, tactical boots and knee guards were either destroyed or dislodged, offering minimal protection. These findings demonstrate a consistent and characteristic injury pattern associated with landmine explosions, predominantly affecting the lower half of the body and resulting in rapid death due to traumatic hemorrhage.

Injury pattern analysis

In the evaluation of six blast-related fatality cases, primary blast injuries were notably absent, with no evidence of barotrauma to air-filled organs such as the lungs, tympanic membranes, or gastrointestinal tract, findings consistent with victims exposed in open or semi-enclosed environments where pressure waves dissipate. In contrast, secondary blast injuries were consistently observed across all cases, characterized by deeply embedded particulate matter, including dirt particles, glass splinters, and cloth fragments within lacerated wounds, indicative of high-velocity projectiles generated by the blast. Case 6 also demonstrated gunpowder residue, considered a secondary injury due to its particulate and penetrative nature.

Tertiary injuries, evident as extensive long-bone fractures, limb avulsions, and abdominal evisceration, reflected high-energy blunt trauma from bodily displacement or structural collapse. Additionally, quaternary injuries were universally present, involving singeing of hair, blackening of skin, and environmental contamination (e.g., embedded dirt and debris), signifying thermal and chemical exposure not explained by direct mechanical trauma.

This consistent pattern, marked by the predominance of secondary and tertiary injuries, coupled with quaternary features, highlights the external and mechanical nature of these insults. The absence of internal barotrauma reinforces the open-space context of these events (Table 3). All six cases exhibited concurrent injury types across all three blast categories, emphasizing the multifactorial trauma mechanisms involved in open-air explosive events.

**Table 3:** Injury classification across blast-related fatalities: primary to quaternary trauma analysis

Case No.	Primary	Secondary	Tertiary	Quaternary
1	Absent	Dirt particles deeply embedded in lacerated wounds	Distal limb separation, extensive fractures	Blackening, singeing of hair, dirt contamination
2	Absent	Glass splinter, dirt particles deeply embedded in lacerated wounds	Extensive limb trauma, separation	Singeing, dirt contamination
3	Absent	Cloth fragment, dirt particles deeply embedded in lacerated wounds	Multiple long-bone fractures	Singeing, dirt contamination
4	Absent	Dirt particles deeply embedded in lacerated wounds	Fractures, limb segment loss	Singeing, dirt contamination
5	Absent	Cloth fragment, dirt particles deeply embedded in lacerated wounds	Limb separation, blunt trauma	Dirt contamination
6	Absent	Gunpowder residue, cloth fragment, dirt particles deeply embedded in lacerated wounds	Evisceration, limb separation	Singeing, dirt contamination

## Discussion

This forensic analysis provides an autopsy-based window into the injury spectrum associated with landmine blast fatalities among security personnel engaged in counter-insurgency operations in the eastern Indian state of Jharkhand. The investigation presents an opportunity to characterize blast-related trauma in the context of asymmetric warfare, where standard military infrastructure and trauma support systems are often compromised. Through methodical examination of six fatal cases, this study highlights the severe injury patterns associated with buried explosive devices, particularly their predilection for producing catastrophic lower-limb trauma and multiregional polytrauma.

The decedents, young, physically fit men aged between 27 and 36, represent the typical operational profile of frontline anti-insurgency forces in India. Their deaths occurred in remote, forested terrains characterized by rugged topography, minimal visibility, and inadequate logistical support, factors that collectively create an environment conducive to the covert emplacement and effective detonation of landmines. The use of pressure-activated or command-detonated IEDs in such contexts reflects not only tactical sophistication but also the strategic intent to maximize lethality through terrain exploitation and delayed evacuation [10,11].

A cardinal finding of this study is the dominance of secondary and tertiary blast injuries, with primary blast effects conspicuously absent. Secondary blast injuries, caused by high-velocity projectiles, were universal and included lacerations with deeply embedded particulate matter. The retrieval of foreign bodies such as gunpowder residues, splinters, and embedded soil particles from muscle planes and body cavities is consistent with high-velocity fragment dispersion, a hallmark of buried explosives. These findings align with injury patterns reported from modern theaters of combat in Iraq and Afghanistan, where similar mechanisms prevail [12,13].

Tertiary blast injuries, resulting from bodily displacement due to the blast wave and subsequent impact, were also prominent. Multiple victims exhibited comminuted fractures of long bones, traumatic amputations, and abdominal evisceration. The explosive force's vertical alignment, which is characteristic of anti-personnel landmines, leads to injuries that are mainly focused in the lower body and pelvic zone, contrasting with torso-level IEDs that generate more general injuries impacting the thoracic and cranial regions [14,15]. This directional injury pattern may assist in forensic reconstruction, particularly in determining blast proximity, victim positioning, and detonation axis.

Quaternary injuries, including singeing of hairs due to thermal burns, epidermal blackening, and contamination by dirt and debris, were consistently documented. These injuries, indicative of close-range thermal exposure, are suggestive of close proximity to the blast, as described in prior literature. The presence of soot and, in one case, gunpowder residue supports immediate proximity to the blast origin and is consistent with scenarios such as command detonation or contact-triggered devices [16].

Conversely, primary blast injuries were notably absent. The lack of such injuries may be attributable to an open-air detonation environment, where rapid atmospheric dispersion of the blast wave mitigates internal pressure effects. This finding is consistent with existing literature on blast physiology, which emphasizes the diminished likelihood of primary blast injury in non-confined settings [17,18].

Despite the use of standard PPE, including ballistic helmets, body armor, and tactical boots, the lower extremities remained inadequately protected against the upward-directed blast forces. All casualties, with the exception of one, experienced a minimum of one traumatic amputation of the lower limb, often bilateral, accompanied by significant vascular disruption and extensive soft-tissue damage. These observations suggest a potential vulnerability within the prevailing protective arrangements, which predominantly concentrate on the torso. The existing body of literature stemming from North Atlantic Treaty Organization (NATO) operations similarly documents enduring morbidity and mortality associated with lower-limb injuries, notwithstanding considerable advancements in helmet and vest design [19]. This highlights the potential need to consider broader protective strategies encompassing the lower body, particularly in environments where underbody blasts constitute a principal threat vector.

A significant forensic theme emerging from this study is the concept of the “lower-limb trauma triad,” comprising traumatic amputation, embedded environmental debris, and rapid exsanguination [20]. This triad, observable in all six cases, represents a consistent pattern within this series and may serve as a preliminary forensic indicator of buried landmine injuries. Its recognition may assist forensic pathologists in establishing the mechanism and manner of death, especially in fragmented or mass-casualty scenarios. Moreover, the presence of this triad may aid in differential diagnoses during postmortem examinations, helping distinguish blast injuries from other forms of traumatic fatalities in conflict zones, as the simultaneity of penetrating, blunt, and thermal forces complicates forensic interpretation [21]. However, this observation is based on a limited sample size and requires validation in larger studies.

From an operational standpoint, the injury patterns documented may reflect factors such as delayed medical intervention, inadequate field trauma training, and logistical barriers to evacuation. Field-level trauma response in counter-insurgency zones remains rudimentary, often lacking hemorrhage control tools such as tourniquets, hemostatic agents, or portable airway devices. This systemic gap is comparable to broader challenges faced in low-resource conflict settings globally, where the “platinum 10 minutes” for trauma intervention remains an unachievable ideal. The integration of Tactical Combat Casualty Care (TCCC) principles into pre-deployment training modules may help reduce preventable deaths [22,23].

In the context of the consistent and catastrophic injury patterns observed in this study, the medico-legal autopsy emerges as a vital tool not only for determining the cause and manner of death but also for reconstructing the dynamics of landmine blast events in counter-insurgency operations. The classification of injuries into primary, secondary, tertiary, and quaternary categories provides a systematic framework for analyzing complex trauma, facilitating accurate medico-legal documentation, and supporting institutional processes related to compensation and operational accountability. When correlated with field variables, such as explosive placement, terrain, and protective equipment, these findings may provide insights into the mechanisms of injury and the operational vulnerabilities of deployed personnel. This supports the importance of continued interdisciplinary engagement between autopsy surgeons, trauma specialists, and tactical planners to refine both the understanding and mitigation of blast-related fatalities.

Limitations

This study offers vital perspectives; however, its interpretative strength is constrained by several limitations. The small sample size limits the broader generalizability of the findings. Additionally, as all cases originated from a single blast event, the findings may be influenced by single-event bias, restricting their applicability across diverse blast scenarios. The retrospective nature of the study introduces inherent selection bias, as only fatal cases subjected to medico-legal autopsy were included. The absence of survivor data further limits comparative analysis between fatal and non-fatal injury patterns, thereby restricting insights into survivability and injury severity gradients. The design of the study also hindered the collection of physiological parameters at the time of injury. Additionally, information regarding the specific type, weight, and configuration of the explosive devices was insufficient, thereby limiting comprehensive correlations between device characteristics and injury severity. Furthermore, the absence of radiological correlation, particularly postmortem computed tomography (PMCT), precluded detailed evaluation of internal injury patterns and foreign body distribution that could complement autopsy findings.

Despite these limitations, the consistent injury patterns observed across cases provide meaningful insights within the defined context. Future investigations should adopt prospective and multidisciplinary approaches, incorporating real-time field data collection, inclusion of survivor cohorts, integration of radiological modalities such as PMCT, and collaboration between autopsy surgeons, trauma specialists, and explosive ordnance experts. Such efforts would enhance the precision of analyses related to injury mechanisms, survivability, and operational risk mitigation.

## Conclusions

This forensic investigation documents severe and recurrent injury patterns observed in landmine blast fatalities in a defined operational context, notably catastrophic lower-limb trauma, deep tissue disruption, and exsanguination. The predominance of secondary, tertiary, and quaternary injuries, together with the absence of primary blast effects, is consistent with an open-air detonation environment and suggests a relative vulnerability of the lower extremities despite the use of standard PPE. The systematic classification of injuries enhances the clarity and precision of medico-legal documentation and provides context-specific insights relevant to forensic interpretation and operational considerations. While these observations arise from a limited dataset, they offer a coherent descriptive account of fatal injury patterns and underscore the important role of medico-legal autopsy in understanding blast-related deaths. These findings may inform hypothesis generation and guide future research, including efforts to refine protective strategies, optimize trauma response systems, and improve forensic documentation in similar asymmetric conflict environments.

## References

[1] UNMAS UNMAS (2015). United Nations Mine Action Service (UNMAS): Landmines, explosive remnants and IED safety handbook. (2015). Accessed: March 11, 2026. Landmines, Explosive Remnants, and IED Safety Handbook.

[2] Zhang S, Han G, Xiong Y, Wang Z, Wang Z, Lai X (2023). Characteristics and mechanism of lower limb injury induced by landmine blast: a research in a rabbit model. Ulus Travma Acil Cerrahi Derg.

[3] Karger B (2014). Forensic ballistics: injuries from gunshots, explosives and arrows. Handbook of Forensic Medicine.

[4] Aggarwal A (2021). Explosion injuries. Textbook of Forensic Medicine and Toxicology.

[5] DePalma RG, Burris DG, Champion HR, Hodgson MJ (2005). Blast injuries. N Engl J Med.

[6] MCRHRDI MCRHRDI (2026). MCRHRDI. Left wing extremism-a challenge to internal security. https://www.mcrhrdi.gov.in/5th_mesfc2023/week8/LEFT%20WING%20MOVEMENT-CHALLENGES%20AND%20INITIATIVES%20OF%20THE%20GOVERNMENT.pdf.

[7] (2026). South Asia Terrorism Portal (SATP). Maoist Insurgency Fatalities Data (2000-2025). https://www.satp.org/datasheet-terrorist-attack/fatalities/india-maoistinsurgency.

[8] Singleton JA, Gibb IE, Bull AM, Mahoney PF, Clasper JC (2013). Primary blast lung injury prevalence and fatal injuries from explosions: insights from postmortem computed tomographic analysis of 121 improvised explosive device fatalities. J Trauma Acute Care Surg.

[9] Oliva A, Grassi S, Grassi VM (2021). Postmortem CT and autopsy findings in nine victims of terrorist attack. Int J Legal Med.

[10] Revill J (2016). Improvised Explosive Devices: The Paradigmatic Weapon of New Wars. Palgrave Macmillan, London, United Kingdom.

[11] Barker AD (2011). Improvised explosive devices in Southern Afghanistan and Western Pakistan, 2002-2009. Stud Confl Terror.

[12] Polk TM, Martin MJ, Eckert MJ (2017). Dismounted complex blast injury management. Front Line Surgery.

[13] Rankin IA, Nguyen TT, Carpanen D, Clasper JC, Masouros SD (2020). A new understanding of the mechanism of injury to the pelvis and lower limbs in blast. Front Bioeng Biotechnol.

[14] Singh B, Basri MA (2022). Lower limb response to anti-personnel landmine blast explosions: injury assessment and mitigation strategies. Hum Factors Mech Eng Def Saf.

[15] Mannion S, Chaloner E, Serrie A (2006). Acute injury caused by landmines. Pain Med.

[16] Daban JL, Peigne V, Boutonnet M, Debien B (2013). Lésions par explosion. Lett Med Phys Readapt.

[17] Scott RA (2016). Primary blast lung injury. Blast Injury Science and Engineering.

[18] Clasper J, Edwards D (2022). Blast injury mechanism. Blast Injury Science and Engineering.

[19] Ramasamy A, Masouros SD, Newell N (2011). In-vehicle extremity injuries from improvised explosive devices: current and future foci. Philos Trans R Soc Lond B Biol Sci.

[20] Singleton JA, Gibb IE, Bull AM, Clasper JC (2014). Blast-mediated traumatic amputation: evidence for a revised, multiple injury mechanism theory. J R Army Med Corps.

[21] Mannion S, Chaloner E (2006). Landmines and landmine injuries: an overview. Pain Med.

[22] Butler FK Jr, Hagmann J, Butler EG (1996). Tactical combat casualty care in special operations. Mil Med.

[23] Conyers K, Gillies AB, Sibley C, McMullen C, Remley MA, Wence S, Gurney JM (2023). Where there's a war, there's a way: a brief report on tactical combat casualty care training in a multinational environment. J Spec Oper Med.

